# Associations of Physical Activity, Sports Participation and Active Commuting on Mathematic Performance and Inhibitory Control in Adolescents

**DOI:** 10.1371/journal.pone.0146319

**Published:** 2016-01-04

**Authors:** Sidsel L. Domazet, Jakob Tarp, Tao Huang, Anne Kær Gejl, Lars Bo Andersen, Karsten Froberg, Anna Bugge

**Affiliations:** 1 Department of Sports Science and Clinical Biomechanics, Division of Exercise Epidemiology, Centre of Research in Childhood Health, University of Southern Denmark, Odense, Denmark; 2 Department of Physical Education, Shanghai Jiao Tong University, Shanghai, China; 3 Department of Sport Medicine, Norwegian School of Sport Sciences, Oslo, Norway; University of Westminster, UNITED KINGDOM

## Abstract

**Objectives:**

To examine objectively measured physical activity level, organized sports participation and active commuting to school in relation to mathematic performance and inhibitory control in adolescents.

**Methods:**

The design was cross-sectional. A convenient sample of 869 sixth and seventh grade students (12–14 years) was invited to participate in the study. A total of 568 students fulfilled the inclusion criteria and comprised the final sample for this study. Mathematic performance was assessed by a customized test and inhibitory control was assessed by a modified Eriksen flanker task. Physical activity was assessed with GT3X and GT3X+ accelerometers presented in sex-specific quartiles of mean counts per minute and mean minutes per day in moderate-to-vigorous physical activity. Active commuting and sports participation was self-reported. Mixed model regression was applied. Total physical activity level was stratified by bicycling status in order to bypass measurement error subject to the accelerometer.

**Results:**

Non-cyclists in the 2^nd^ quartile of counts per minute displayed a higher mathematic score, so did cyclists in the 2^nd^ and 3^rd^ quartile of moderate-to-vigorous physical activity relative to the least active quartile. Non-cyclists in the 3^rd^ quartile of counts per minute had an improved reaction time and cyclists in the 2^nd^ quartile of counts per minute and moderate-to-vigorous physical activity displayed an improved accuracy, whereas non-cyclists in the 2^nd^ quartile of counts per minute showed an inferior accuracy relative to the least active quartile. Bicycling to school and organized sports participation were positively associated with mathematic performance.

**Conclusions:**

Sports participation and bicycling were positively associated with mathematic performance. Results regarding objectively measured physical activity were mixed. Although, no linear nor dose-response relationship was observed there was no indication of a higher activity level impairing the scholastic or cognitive performance.

## Introduction

Evidence has established habitual physical activity (PA) to have a wide range of beneficial effects on physiological [1] as well as psychological [2] health compounds such as blood pressure, the metabolic syndrome, bone mineral density, and self-esteem in children and youth. PA has further been associated with better cognitive performance and improved brain structures and functioning in elderly and cognitively impaired subjects [3,4]. A growing body of evidence suggests similar beneficial effects on the developing brain [5]. Specifically, aerobically fit children have demonstrated increased activity in the prefrontal cortex relative to lower fit children prior to stimulus presentation during a modified Eriksen flanker task indicating stronger proactive inhibition [6]. Whether these relationships are directly transferable to adolescents is questionable due to lack of investigations conducted in the adolescent population and the potential influence of changing sex hormonal status during puberty on neuroplasticity [7]. So far the majority of studies has been conducted in pre-adolescent children and has mainly relied on self-reported PA measures due to higher feasibility [8]. In children, gains in mental functioning due to exercise are mostly observed on tasks that involve executive functions [9], which comprise working memory, cognitive flexibility and inhibitory control [10]. The latter is involved in planning and selecting strategies that organize goal-directed actions [11] and is superior to basic information processing such as; encoding, stimulus evaluation, response selection and response execution [12]. Inhibitory control during preschool has been found to be a prominent correlate of early mathematic and reading ability in kindergarten suggesting that superior self-regulation skills are a predictor of school readiness and scholastic performance [13]. There is increasing evidence of a strong relationship between executive functions and children’s academic performance, especially, mathematics achievement [14].

A few studies have investigated the association of objectively measured PA on cognitive and scholastic performance in adolescents [15–18], but the results have been mixed. To our knowledge this is the first study to address the relationship between cognition and kinesiology in adolescence by combining objective measures of habitual PA and self-reported PA behaviors.

The aim of this study was to examine the associations of objectively measured PA, self-reported active commuting to school (bicycling or walking) and organized sports participation in relation to mathematic performance and inhibitory control in adolescents. We hypothesized that a higher PA level, active commuting especially bicycling, and engagement in sports may be associated with better mathematic performance and superior inhibitory control.

## Methods

### Participants

This cross sectional study was based on baseline data from the cluster-randomized controlled trial LCoMotion–Learning, Cognition and Motion. Rationale, design and methods for the study have previously been published [19] and only measures pertinent to this study are described here. Data was collected in November and December 2013 at fourteen schools from the five main regions of Denmark. Eleven schools were recruited by an external collaborator and the last three were recruited from existing networks. All students from 6^th^ and 7^th^ grade (12–14 years) were invited to participate in the study (n = 869) of which 87% gave written consent to participate (n = 759). The final sample comprised 568 students, who had complete data on both outcome variables, all covariates applied in the models and at least one of the three exposure variables. Testing was carried out in one day at each school from 08.00 to 14.00 except objectively measured PA, which was gathered a week during November or December 2013. Parents gave written informed consent prior to their child’s participation. This study was carried out in accordance with the Helsinki Declaration and approved by The Regional Scientific Ethical Committees for Southern Denmark (S-20130104).

### Outcome variables

Inhibitory control was assessed by a modified Eriksen flanker task [20]. The task was a computer-based version consisting of five white arrows on a black screen. The student was asked to respond to the target arrow in the middle. An arrow pointing to the right “>” required a right button response on the keyboard as well as a left arrow “<” required a left button response. The two flanker arrows on each side of the target arrow worked as distractors and would point in either same direction “>>>>>” (congruent trial) or opposite direction “<<><<” (incongruent trial) as the target arrow. E-Prime 2.0 (Psychology Software Tools Inc., Sharpsburg, PA) was used for modifying and running the flanker task. Each stimulus was presented for 120 milliseconds (ms) and the student was to respond within 200 to 1470 ms post onset of stimuli in order to perform a valid response. The task consisted of 2 x 75 trials with an equal number of congruent and incongruent trials presented in a random order. Preferably, students were tested alone in a quiet room. However, due to large school classes students were often tested in a room with few others. Instructions were given verbally by a trained staff member prior to testing. The results were presented in two domains; accuracy (percentage of right responses) and reaction time (number of ms on right responses). Students, who achieved ≤ 50% on response accuracy, were excluded from the analysis. The incongruent condition is found to cause a delay in reaction time as well as a decrease in accuracy relative to the congruent condition, due to an increased inhibitory load elicited by the distracting flankers [20]. Therefore, the results were presented as an interference score between the congruent and incongruent condition; reaction time (incongruent—congruent) and accuracy (congruent—incongruent) in order to optimize the measure of inhibitory control by removing the variation caused by less cognitively demanding processes such as; facilitation of attention and perception. However, means in reaction time and accuracy during congruent and incongruent trials are presented under descriptive statistics of participants.

Mathematic performance was derived from a custom-made mathematics test adjusted to the two year groups (6^th^ and 7^th^ grade). The test was correlated with a national standardized mathematics test by the Danish Ministry of Education (correlation coefficient 0.87). Likewise, test-retest reliability was carried out on 66 participants with an excellent correlation (0.92). Students were tested in the class room for 45 minutes under teacher or research staff supervision. The test was completed individually and no aids were permitted. The test comprised fifty questions within different domains of arithmetic and arithmetic problem solving. Research staff subsequently scored the test. The final score was the percentage of correct answers.

### Exposure variables

PA was assessed objectively using hip-mounted GT3X and GT3X+ accelerometers (Actigraph, Pensacola, FL, USA). The students were instructed to wear the monitor during waking hours for at least eight consecutive days. Monitors were distributed on Mondays or Tuesdays during November and December 2013. Monitor type was randomly selected at student level. In order to eliminate reactivity bias the monitors started recording the day after distribution. Sample epoch length was set to 2 seconds for GT3X and 30 Hz for GT3X+, but subsequently downloaded in 10 seconds epochs. A sequence of more than 30 minutes of consecutive zeros was considered non-wear time and eliminated from the analysis. In order to account for students who failed to remove the monitor at night, only activity recorded during 06.00 and 22.00 was analyzed. A valid measurement was attained by a minimum of four days with at least 10 hours of daily recorded activity. According to Trost and colleagues four days of monitoring are required in order to achieve a reliability of 0.80 with this age group [21]. Internally developed software, Propero (University of Southern Denmark, Odense, Denmark), was used to reduce data for further analysis. Only the vertical axis was analyzed due to better comparability between accelerometer outputs. PA was expressed as mean CPM and mean minutes per day in MVPA. Intensity cut-off points by Evensson and colleagues [22] divided by 1.5 were used to define MVPA.

Information regarding transportation to school was obtained prior to the flanker task, where a research staff member asked how the student arrived to school that day. Response categories comprised six options (bicycling, walk, car, bus, train, others) but were merged into three categories 1) car/public transport, 2) walk and 3) bicycling for analysis. Information on habitual transport mode to and from school was gathered as well from a student questionnaire using the same response categories as abovementioned. Due to more missing observations in the student questionnaire the response from the test day was used for analysis. However, the agreement between the written response from the student questionnaire and the verbal response on the test day was good (0.75). Furthermore, test-retest reliability of the written question and the verbal question displayed an excellent (0.93) and good agreement (0.76), respectively.

Information regarding sports participation was gathered from the student questionnaire given to the school teachers, who were then responsible for going over the questions with the class within a week after being tested. In order to accommodate students with difficulties in literacy the teacher would read the questions out aloud and give the students time to answer each question individually. The question regarding sports participation comprised five categories; 1) participate in organize sports several times a week and train hard, 2) participate in organized sports approximately one time a week and active on a daily basis, 3) do not participate in organized sports but otherwise active, 4) participate in activities just not related to sports or PA 5) prefer sedentary activities such as TV viewing, gaming etc. Students were defined as participating in organized sports if they had responded to option 1) or 2). Test-retest reliability of the question regarding sports participation displayed a good agreement (0.75).

### Covariates

Height was measured with a stadiometer to the nearest 0.5 cm (Seca, Birmingham, UK). Weight was measured on an electronic scale to the nearest 0.1 kg (Tanita, Tokyo, Japan). Waist circumference was measured twice with a measuring tape to the nearest 0.5 cm. An additional measurement was taken if the first two differed more than 2 cm and a mean value of the two closest measures was calculated. Body mass index (BMI) was calculated as weight (kg)/height^2^ (cm) and overweight comprising obesity was defined using age- and sex-adjusted cut points [23]. Pubertal development was assessed using self-report with five depicted developmental stages related to secondary sex characteristics; pubic hair for boys and breast development for girls [24].

Information on special teaching (yes/no) and socioeconomic status (SES) was obtained from a parental questionnaire delivered to the student on the test day or forwarded to the student’s home address by mail. Special teaching was defined as supporting teaching outside the classroom during school hours. The highest completed educational level of the mother or female guardian of the student was used as an indicator of SES with categories comprising 1) no formal education, 2) vocational training or < 3.5 years of adult education and 3) ≥ 3.5 years of adult education. Information on breakfast consumption (yes/no) on the test day was obtained prior to the flanker task by a research staff member. Age was calculated from the child’s birthday and the test day.

### Statistics

Differences in means of exposure and outcome variables between sexes were tested using an unpaired two-sample t-test for continuous variables, a Pearson’s chi^2^ test for binary variables and a Fisher’s exact test for categorical variables. Test-retest reliability of continuous variables was tested with Pearson’s correlation, whereas categorical variables were tested with kappa statistics.

Main outcomes were analyzed using mixed model regression in order to allow for observations being clustered within school classes. The inter-class correlation coefficient (ICC) for the mathematic test was high (16%), whereas the ICC for the flanker task in the incongruent condition accounted for 1% and 4% of the variance in accuracy and reaction time, respectively. In order to accommodate an observed non-linearity in the relationship between CPM or MVPA and mathematic performance or inhibitory control, mean CPM and mean minutes per day in MVPA were divided into sex-specific quartiles. An a priori selection of possible covariates (age, SES, special teaching and pubertal development) was foreseen. Age, SES and special teaching reached statistical significance (p˂0.05) and were adjusted for in the mixed model regression. Results were also adjusted for consumption of breakfast, which has been previously found to be correlated with PA as well as cognitive and scholastic performance [25]. Total PA level (CPM and MVPA) was stratified by bicycling or not-bicycling to school. This stratification was made in order to accommodate information bias due to measurement error subject to the accelerometer as bicycling is known to be highly underestimated during hip-mounted activity monitoring [26].

Assumptions of normality and homoscedasticity of residuals were tested. Data was analyzed using Stata IC 13.0 (StataCorp, College Station, Texas, USA) with an alpha = 0.05 (two-sided).

## Results

### Characteristics of study participants

Characteristics of study participants are presented in Table 1. Compared to girls, boys were on average older, had higher waist circumference and higher mathematic score. On the contrary girls were more mature and had higher accuracy during incongruent trials of the flanker task relative to boys. In total PA and MVPA as well as in organized sports participation boys were more active than girls.

**Table 1:** Characteristics of study participants stratified by sex (n = 568).

	n =	Boys	n =	Girls	p =
Age (year)	269	13.0 (0.6)	299	12.9 (0.6)	0.01*
Body weight (kg)	267	49.6 (10.8)	292	50.4 (10.1)	0.42
Body height (cm)	268	159.9 (8.8)	292	159.8 (7.0)	0.94
BMI (kg/m^2^)	267	19.3 (3.1)	292	19.6 (3.3)	0.18
Waist circumference (cm)	267	72.4 (8.6)	291	69.0 (8.7)	<0.01*
Overweight (%)	267	13.9	292	16.4	0.40
Tanner stages (n)	262	11/65/110/62/14	286	1/13/149/108/15	<0.01*
SES (n)	269	49/122/98	299	37/140/122	0.14
Special teaching (%)	265	9.8	297	6.7	0.18
Breakfast consumption (%)	269	91.5	299	89.0	0.32
Mathematic score (%)	269	43.9 (21.2)	299	39.4 (19.2)	0.01*
Reaction Time—congruent (ms)	269	458.4 (72.1)	299	466.8 (65.0)	0.15
Accuracy—congruent (%)	269	95.0 (6.3)	299	94.8 (6.5)	0.73
Reaction Time—incongruent (ms)	269	537.5 (85.9)	299	547.1 (83.1)	0.18
Accuracy—incongruent (%)	269	80.5 (12.7)	299	84.2 (10.9)	<0.01*
Total PA (cpm)	145	436.1 (142.2)	200	394.7 (131.6)	0.01*
Total MVPA (minutes/day)	145	53.5 (21.4)	200	45.2 (15.6)	<0.01*
Organized sports participation (%)	243	82.31	279	74.9	0.04*
Transport mode (n)	268	106/63/99	299	138/52/109	0.13

BMI; Body Mass Index. Overweight; comprising obesity. Tanner stages; comprising five developmental stages corresponding to secondary sex characteristics (stage 1 indicating immaturity/stage 2–4 indicating degrees of maturation/stage 5 indicating maturity). SES; Socio-Economic Status (comprising no formal education/vocational training or < 3.5 years of adult education/≥3.5 years of adult education). PA; physical activity. MVPA; moderate-to-vigorous physical activity. Transport mode; car or public transport/walk/bicycling. All values are crude means with standard deviations unless stated otherwise.

* Two-sided alpha ≤0.05 indicating a significant difference between sexes.

### Associations with total PA level

Cyclists and non-cyclists did not differ in estimated mean CPM (p = 0.27), however non-cyclists had a higher estimated mean MVPA relative to cyclists (p = 0.03) (data not shown). Results from mixed model regression regarding total PA level (Table 2) showed a higher mathematic score among non-cyclists in the 2^nd^ quartile of CPM and cyclists in the 2^nd^ and 3^rd^ quartile of MVPA relative to the least active quartile (the 1^st^ quartile). A similar trend was found for higher mathematic score with non-cyclists in the 2^nd^ and 3^rd^ quartile of MVPA and for cyclists in the 4^th^ quartile of MVPA. However, these differences only reached borderline significance (p = 0.07 and p = 0.06, respectively).

**Table 2:** Results from mixed model regression with total physical activity level stratified by bicycling status.

**Mathematic score (%)**						
	**Cyclists (n = 148)**			**Non-cyclists (n = 193)**		
**CPM quartiles**	β	95% CI	P	β	95% CI	P
1^st^	reference	-	-	reference	-	-
2^nd^	4.0	-3.8;11.8	0.31	7.4	0.2;14.7	0.04*
3^rd^	7.4	-1.1;15.9	0.09	5.1	-1.8;12.0	0.15
4^th^	2.9	-6.1;11.8	0.53	3.8	-3.2;10.7	0.29
**MVPA quartiles**						
1^st^	reference	-	-	reference	-	-
2^nd^	12.4	5.1;19.8	<0.01*	6.7	0.6;14.0	0.07
3^rd^	8.1	0.3;15.9	0.04*	6.9	-0.5;14.2	0.07
4^th^	8.3	-0.3;16.9	0.06	4.2	-3.0;11.4	0.25
**Interference score on reaction time**						
**CPM quartiles**						
1^st^	reference	-	-	reference	-	-
2^nd^	-1.4	-18.3;15.5	0.87	-11.3	-24.6;2.0	0.10
3^rd^	-4.3	-22.5;14.0	0.65	-13.6	-26.4;-0.8	0.04*
4^th^	9.2	-28.1;9.8	0.34	-8.3	-21.0;4.4	0.20
**MVPA quartiles**						
1^st^	reference	-	-	reference	-	-
2^nd^	7.6	-8.8;24.0	0.37	-4.3	-17.8;9.2	0.54
3^rd^	6.1	-11.2;23.3	0.49	-7.9	-21.5;5.8	0.26
4^th^	-7.9	-26.6;10.8	0.41	-6.9	-20.0;6.3	0.31
**Interference score on accuracy**						
**CPM quartiles**						
1^st^	reference	-	-	reference	-	-
2^nd^	-4.3	-8.4;-0.33	0.03*	2.3	-1.4;6.0	0.22
3^rd^	-3.0	-7.3;1.4	0.18	-0.4	-4.0;3.1	0.81
4^th^	-2.1	-6.7;2.5	0.37	0.9	-2.6;4.4	0.61
**MVPA quartiles**						
1^st^	reference	-	-	reference	-	-
2^nd^	-3.9	-7.8;-0.0	0.05*	4.9	1.3;8.6	0.01*
3^rd^	-3.8	-7.9;0.3	0.07	1.1	-2.6;4.8	0.56
4^th^	-2.2	-6.8;2.3	0.33	3.1	-0.5;6.6	0.10

CPM; counts per minute. MVPA; moderate-to-vigorous physical activity. Results were adjusted for age, sex, socio-economic status, special teaching and breakfast consumption.

* Two-sided alpha ≤0.05 indicating a significant difference from the reference group.

Results relating to inhibitory control showed that the flanker task functioned as intended. Mean reaction time was higher and mean accuracy was lower during incongruent trials (545.7 ms and 82.3%) compared to during congruent trials (464.7 ms and 94.8%) indicating a greater inhibitory load under incongruent conditions. A lower interference score indicates less difference between congruent and incongruent responses corresponding to an improved inhibitory control.

Only non-cyclists in the 3^rd^ quartile of CPM were found to have a significantly lower interference score on reaction time compared to the least active quartile. Referring to the interference score on accuracy cyclists in the 2^nd^ quartile of CPM and MVPA displayed a significantly lower interference score relative to the reference group. As opposed to this non-cyclists in the 2^nd^ quartile of MVPA displayed a significantly higher interference score relative to those in the 1^st^ quartile.

### Associations with organized sports participation and active commuting

Results from mixed model regression in relation to organized sports participation and active commuting (Table 3) demonstrated a superior mathematic performance with engagement in sports versus no engagement in sports and bicycling to school compared to passive transport. On the contrary, results regarding inhibitory control showed no associations. Of those who claimed to bicycle, walk or use car or public transportation to school, 82%, 75% and 76% claimed to participate in organized sports, respectively (data not shown).

**Table 3:** Results from mixed model regression with organized sports participation and active commuting.

** **	**Mathematic score (%)**		
**Organized sports (n = 517)**	β	95% CI	P
No	reference	-	-
Yes	8.0	4.2;11.8	<0.01*
**Transport mode (n = 561)**			
Car/public transport	reference	-	-
Walk	0.1	-4.1;4.4	0.95
Bicycle	5.4	1.9;8.8	<0.01*
	**Interference score on reaction time**		
**Organized sports (n = 517)**			
No	reference	-	-
Yes	-3.6	-11.3;4.1	0.36
**Transport mode (n = 561)**			
Car/public transport	reference	-	-
Walk	1.4	-6.9;9.8	0.74
Bicycle	4.0	-2.7;10.6	0.24
	**Interference score on accuracy**		
**Organized sports (n = 517)**			
No	reference	-	-
Yes	-1.9	-3.8;1.1	0.07
**Transport mode (n = 561)**			
Car/public transport	reference	-	-
Walk	-0.5	-2.7;1.6	0.64
Bicycle	-0.1	-1.8;1.7	0.93

Results were adjusted for age, sex, socio-economic status, special teaching and breakfast consumption.

* Two-sided alpha ≤0.05 indicating a significant difference from the reference group.

## Discussion

Our results showed a clear association between organized sports participation and active commuting in relation to bicycling to school and a superior mathematic performance. Results regarding total PA level as exposure were mixed.

We did not find a positive linear relationship with total PA level and mathematic performance or inhibitory control as hypothesized. Nor did we observe a dose-response relationship in the associations. A Dutch study in adolescents found MVPA and scholastic performance to follow an inverted u-shaped relationship, although these associations were moderated by academic year, volume and intensity of PA and school grade [18]. Our few significant findings generally indicated that the least active subjects were outperformed by their more active peers in mathematics with the same trend observed for cyclists in interference score on accuracy, although there was no indication of further benefit of being in the highest end of the activity scale. Esteban-Cornejo and colleagues also reported consistently lower grade points in mathematics, language, and lower total grade point average in children and adolescents, who were most active, although this association was weak and became non-significant among quartiles of objectively measured PA [27].

The assumption of lower scholastic and cognitive performance among highly active subjects might not be causally related to a high total PA level per se. It is rather plausible that the association appeared by chance. In our case the null finding might be due to lack of statistical power or residual confounding. The latter might, theoretically, cover factors relating to the interests of the highly active adolescents. These subjects might be less interested in studying or less likely to benefit from standard sedentary teaching.

Our results regarding objectively measured PA did not form a clear pattern, though there was no reason to suggest a negative association of PA on cognitive and scholastic performance. The single finding indicating a negative association (non-cyclists’ interference score on accuracy in the 2^nd^ quartile of MVPA) might be explained by multiple comparisons (type one error). Other studies, which have addressed the relationship between objectively measured PA and cognitive functions, have encountered similar inconsistent findings. Booth and colleagues [15] did not report results to be uniform across all cognitive tasks (attention and executive attention), suggesting that there might be selective benefits of PA. Though, they found MVPA may be beneficial for attention processes in adolescents, especially in males. Syväoja and colleagues [17] found high levels of MVPA to be associated with superior performance in a reaction time test (Reaction Time, RTI), whereas high levels of sedentary time were associated with superior performance in a sustained attention task (Rapid Visual Information Processing, RVP). However, objectively measured MVPA and sedentary time were not associated with other measures of cognitive functions (visual memory, executive functions and attention).

Our findings occurring in relation to organized sports participation and active commuting on mathematic performance were convincing. This might suggest that benefits of PA are setting-specific. It might be a matter of the context or quality of the PA, rather than just the frequency or intensity. Within the organized sports participation implies elements of muscle strength, flexibility, and motor skills besides a social component, which are not captured by the accelerometer. All individual elements comprised in sports participation might have mediated the association on mathematic performance. In respect to active commuting, bicycling has been found to be highly associated with aerobic fitness in Danish children and adolescents [28]. From this point of view, it is plausible that bicycling to school may have had a spillover effect on leisure time bicycling.

If this has been the case, bicycling has impacted the total MVPA level hugely without affecting the objectively measured PA estimates. Therefore, the observed difference in estimated MVPA in favor of non-cyclists must be interpreted with caution.

However, a Dutch study in adolescents found active commuting to school to be positively associated with executive functioning, although this association was only evident for girls [29]. Furthermore, parallel cross-sectional data on aerobic fitness from the LCoMotion study found higher aerobic fitness to be associated with indicators of improved inhibitory control and superior mathematic performance [30].

### Strengths and limitations

A clear strength to this study was the objective measurement of the total PA level in combination with self-reported information on organized sports participation and active commuting. Accelerometry is preferable to use in children and youth as the method captures most daily activities including non-exercise ambulatory movement while bypassing recall bias and systematic overestimation of the activity level [31]. Unfortunately, some activities such as bicycling, weight-bearing and upper-body exercises are underestimated and water-based activities such as swimming are undetectable [21]. In order to meet with these constraints results were stratified by bicycling status as biking accounts for a considerably large amount of daily unmeasured MVPA when using accelerometry [26]. Information on sports participation was intended for including activities not registered by the accelerometer. Besides, the total sample size was fairly large (n = 568), which improves the external validity.

The most predominant limitation was the cross-sectional design. Although the matter of causality cannot be replied, these results may form the basis of generating new hypotheses in the relationship between PA and cognition and scholastic performance in adolescents. Another limitation was the varying sample sizes due to different sources of exposure. Especially, the inclusion criteria of four days of accelerometer-registered activity lowered the response rate. However, this criterion was selected to retain a high reliability. Information on sports participation was derived from a question regarding leisure time activities and was not referring to sports participation exclusively, which might have introduced misclassification to some extent. Inherently, self-report data is subject to information bias such as recall bias, response bias and misclassification. However, the displayed test-retest reliability of the student questionnaire was acceptable. Having solely one measure of inhibitory control and scholastic performance was a limiting factor as well. To enhance the outcome measures a more complete cognitive test battery including the other core executive functions (working memory and cognitive flexibility) besides focusing on more subject-specific skills (e.g. reading, literacy, spelling, problem solving, creative thinking) may be preferable. However, due to resource restraint only one measure within the cognitive and scholastic domain was chosen, respectively. Inhibitory control and mathematic performance was selected based on evidence specifically linking inhibitory control with early mathematic ability [13]. In detail, the largest effects of exercise training were found for executive functions in both children [9] and elderly [3] with mathematic performance being significantly correlated with various measures of executive functioning in children [32]. Furthermore, higher fit children have displayed better mathematics achievement in relation to cortical gray matter thinning during brain maturation compared to lower fit children [33].

Due to resource restraint in the school-setting students performed the flanker task in rooms with few other students present. This might have obstructed the validity. However, the risk of misclassification on this behalf was considered to be non-differential. Besides, the students were always supervised by research staff securing a quite environment.

## Conclusions

Results showed a consistently positive association of organized sports participation and bicycling to school on mathematic performance. Although, no linear nor dose-response relationship was observed between objectively measured PA and the outcomes, there was no indication of a higher activity level impairing the scholastic or cognitive performance.

Taking the study design into account further research is needed in order to comprehend the interplay between cognition and kinesiology in adolescence. It is recommended that future research target a broader understanding of PA, cognition and scholastic performance by including different activity behaviors, more aspects of cognitive functioning and varied scholastic learning objectives. Concisely, robust experimental research is required in order to determine the frequency, intensity and type of PA most suitable for optimizing adolescents’ scholastic performance and tapping the individual cognitive potential.
